# CXCL13 chemokine as a promising biomarker to diagnose neurosyphilis in HIV-negative patients

**DOI:** 10.1186/s40064-016-2462-4

**Published:** 2016-06-16

**Authors:** Yan-Li Zeng, Yi-Qiang Lin, Ning-Ning Zhang, Chao-Ning Zou, Hui-Lin Zhang, Feng Peng, Zhao-Ji Liu, Wei-Hong Zheng, Jiang-Hua Yan, Li–Li Liu

**Affiliations:** Center of Clinical Laboratory, Zhongshan Hospital, Medical College Xiamen University, Xiamen, 361004 China; Department of Neurology, Zhongshan Hospital, Medical College Xiamen University, Xiamen, 361004 China; Cancer Research Center, Medical College Xiamen University, Xiamen, Fujian Province 361102 China

**Keywords:** Neurosyphilis, *Treponema pallidum*, CXCL13, Cerebrospinal fluid, Neuroinflammation

## Abstract

**Background:**

Chemokine ligand 13 (CXCL13) is believed to play a role in the recruitment of B cells in the central nervous system during neuroinflammation. Neurosyphilis is a group of clinical syndromes of the central nervous system caused by *Treponema pallidum* (*T. pallidum*) infection. The relationship between CXCL13 and neurosyphilis still needs further study. In our study, CSF and serum CXCL13 concentrations were detected among 40 neurosyphilis patients, 31 syphilis/non-neurosyphilis patients, 26 non-syphilis/other central nervous system diseases patients. Serum CXCL13 concentrations were detected in 49 healthy persons. All enrolled persons were HIV-negative. Receiver operating characteristic (ROC) analysis was performed to determine the threshold value that could distinguish neurosyphilis from syphilis.

**Results:**

We found that the CSF CXCL13 concentrations and CXCL13 quotient (Q_CXCL13_) were significantly increased in neurosyphilis patients compared to syphilis/non-neurosyphilis (χ^2^ = 21.802, *P* < 0.001) and non-syphilis patients (χ^2^ = 7.677, *P* = 0.002). ROC curve analyses revealed that CSF CXCL13 concentrations and Q_CXCL13_ could serve as valuable biomarkers for differentiating neurosyphilis from non-neurosyphilis/syphilis.

**Conclusions:**

The CSF CXCL13 and Q_CXCL13_ could serve as valuable biomarkers for differentiating neurosyphilis from non-neurosyphilis/syphilis in HIV-negative patients.

## Background

Neurosyphilis is a group of clinical syndromes of the central nervous system caused by *Treponema pallidum* (*T. pallidum*) infection. Syphilitic infection of the central nervous system can occur early or late in the course of the disease (Marra 2009). The clinical manifestations of neurosyphilis are protean, but the classification into asymptomatic, meningeal, meningovascular and parenchymatous disease is helpful and widely used (Friedrich et al. 2009). The diagnosis of neurosyphilis is based on a combination of clinical and laboratory findings. According to the guidelines of the United State Centers for Disease Control and Prevention, the cerebrospinal fluid (CSF) *T. pallidum*-specific antibody and non-treponemal CSF-venereal disease research laboratory (CSF-VDRL) tests can be used as two special markers to diagnose neurosyphilis (Workowski and Berman 2010). However, these tests are not sensitive or specific enough to detect all cases of neurosyphilis, and there is no perfect test to diagnose or exclude neurosyphilis. Because CSF-VDRL is an imperfect “gold standard” for the diagnosis of neurosyphilis, laboratory diagnosis of neurosyphilis usually depends on various combinations of reactive serologic test results, such as CSF cell count and protein, or other markers of disease. Most of the other tests are both insensitive and nonspecific and must be interpreted in relation to additional test results and clinical assessments.

In recent years, CSF CXCL13 concentrations have been suggested as a marker for the diagnosis of neurosyphilis in HIV-infected patients because they are independent of CSF pleocytosis and the markers of HIV (Marra et al. 2010). CXCL13 is produced by antigen-presenting cells and is a selective chemoattractant for B cells and B-helper T cells in different neuroinflammatory diseases. CXCL13 seems to be the major determinant of B cell recruitment to the central nervous system compartment in different neuroinflammatory diseases (Husson et al. 2002; Cadavid and Londono 2009). Elevated CXCL13 levels were found not only in the CSF of neurosyphilis patients with HIV infection but also in the CSF of patients with multiple sclerosis, acute Lyme neuroborreliosis, clinically isolated syndrome and other inflammatory neurological diseases (Kowarik et al. 2012). Here, we assessed the regulatory response of CXCL13 in the CSF and serum of patients with neurosyphilis, syphilis and other central nervous system diseases, as well as healthy volunteers to explore the utility of CXCL13 concentrations in differentiating neurosyphilis from non-neurosyphilis/syphilis in HIV-negative patients.

## Methods

### Study population

A retrospective study was performed in Zhongshan Hospital, Medical College of Xiamen, from September 2011 to March 2014. A total of 146 subjects were included in this study excluded three cases who were found to have syphilis 1 year, 2 years, 8 years ago by outpatient treatment. Forty patients were eligible for enrollment if they had clinical or serological evidence of neurosyphilis. Thirty-one patients with syphilis/non-neurosyphilis (excluded from neurosyphilis) and 26 non-syphilis patients with CSF abnormalities were enrolled in this study, while 49 healthy volunteers were used as controls. The diagnoses of syphilis and neurosyphilis were determined as previously reported (Tong et al. 2013a, 2014). The 26 non-syphilis patients visited the hospital because of neurological symptoms and were ultimately diagnosed with central nervous system diseases by clinical examination, including viral meningitis (8 patients), epileptic seizure (7 patients), spinal demyelination (3 patients), Cryptococcus meningitis (2 patients), peripheral neuropathy (2 patients), Guillain-Barré syndrome (1 patient), multiple sclerosis (1 patient), motor neuron disease (1 patient), and encephalomyelitis (1 patient). All research subjects enrolled into our study were first-visit patients, none of them were treated for syphilis or neurosyphilis before the CSF collection. All participants were negative for HIV. Except for the healthy volunteers, all other participants underwent lumbar puncture according to symptoms or signs. This study was approved by the Institutional Ethics Committee of the Medical College of Xiamen University and complied with national legislation and the Declaration of Helsinki guidelines.

### Laboratory testing

CXCL13, rapid plasma reagin (RPR) and *T. pallidum* particle agglutination (TPPA) assay in serum and CSF for HIV-negative neurosyphilis were conducted at Zhongshan Hospital, Medical College of Xiamen University, from September 2011 to March 2014. All serological tests were performed using the same specimen, and the results of both tests were reported simultaneously. Serologic and CSF tests for syphilis were performed using RPR (Intec, Xiamen, China) and TPPA (Fujirebio, Tokyo, Japan) according to the manufacturer’s instructions and our previous studies (Lin et al. 2010, 2011; Hu et al. 2016; Wu et al. 2012). The concentrations of CXCL13 in the serum and CSF were measured using human CXCL13/BLC/BCA-1 ELISA Kits (CUSABIO, Wuhan, China), according to the manufacturer’s instructions. CSF protein, CSF and serum albumin and CSF white blood cell (WBC) examinations were performed according to the manufacturer’s instructions and our previous studies (Liu et al. 2013a). HIV infection was excluded in all patients with electrochemiluminescence immunoassay (ECLIA) using HIV 1 + 2 antigens/antibodies (F. Hoffmann-La Roche Ltd., Basel, Switzerland). The albumin quotient (Q_alb_) was calculated using the following formulae: Q_alb_ = (Albumin_CSF_/Albumin_Serum_) × 10^−3^. All patients with impaired blood-CSF barriers (defined as albumin quotient [(Albumin_CSF_/Albumin_Serum_) × 10^−3^ ≥ 8.0] showed higher chemokine/cytokine levels in the CSF than patients with intact barriers (Kowarik et al. 2012). The CXCL13 quotient (Q_CXCL13_) was calculated using the following formulae: Q_CXCL13_ = [(CXCL13_CSF_/Albumin_CSF_)/(CXCL13_Serum_/Albumin_Serum_)] (Kowarik et al. 2012; Stilund et al. 2014). The TPPA index was calculated according to the following formula: CSF TPPA/(CSF_albumin_ × 10^3^/serum_albumin_) (Mothapo et al. 2015).

### Diagnostic criteria

Syphilis was diagnosed using serological data, personal/medical history and clinical characteristics, according to US CDC (Workowski and Berman 2010) and ECDC guidelines (Janier et al. 2014), as previously reported (Tong et al. 2014; Lin et al. 2010, 2011; Hu et al. 2016; Wu et al. 2012). The criteria for neurosyphilis diagnosis were described in a previous study (Tong et al. 2013a, 2013b; Mothapo et al. 2015; Liu et al. 2012).

### Statistical analysis

All statistical analyses were performed using SPSS version 17.0 (SPSS, Chicago, IL, USA). Normality of the distribution of continuous variables was tested by the Shapiro–Wilk test. Kruskal–Wallis *H* test was used for continuous variables with skewed distribution to determine differences among groups. The Nemenyi test was performed for multiple comparisons. The association among CXCL13 concentrations, clinical parameters, and CSF laboratory abnormalities was analyzed using the Spearman rank correlation test. The sensitivity, specificity, and area under the curve (AUC) for CSF CXCL13 concentrations, the Q_CXCL13_, and serum CXCL13 concentrations were determined using receiver operator characteristic (ROC) analysis (Zajkowska et al. 2011). *P* < 0.05 was considered statistically significant.

## Results

### Characteristics of the study subjects

**Fig. 1:**
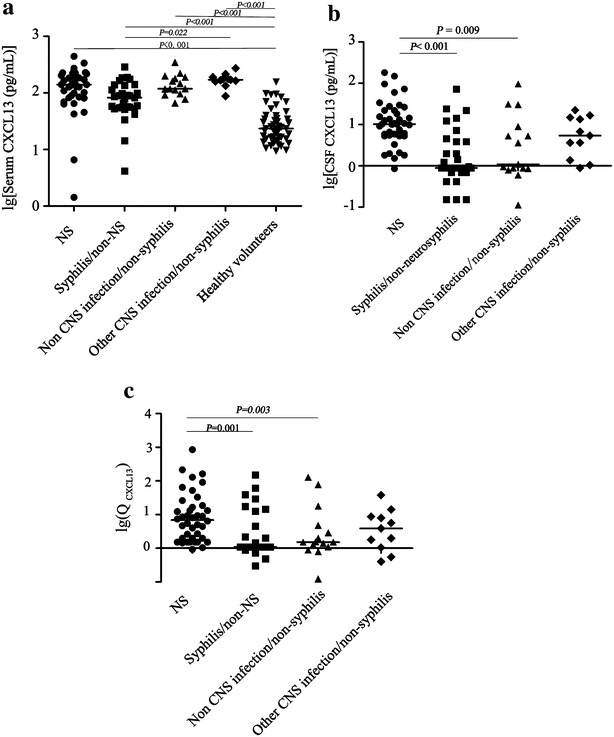
Scatterplot of serum CXCL13 concentrations (**a**), CSF CXCL13 concentrations (**b**) and Q_CXCL13_ (**c**) in different groups which was used a logarithmic scale for the Y-axis. **a** The concentrations of CXCL13 in serum were statistically different among the five groups. The concentrations in the healthy group were statistically lower than the other four groups (*P* < 0.001). The concentrations in syphilis/non-neurosyphilis were statistically lower than non-syphilis/other CNS infection group (*P* = 0.022). The concentrations in neurosyphilis group were higher than syphilis/non-neurosyphilis group, but there was no statistical difference (*P* = 0.084). **b** The concentrations of CXCL13 in CSF were statistically different among the four groups. The concentrations in the neurosyphilis group were statistically higher than the syphilis/non-neurosyphilis (*P* < 0.001), and non-syphilis/non CNS infection group (*P* = 0.009). **c** The Q_CXCL13_ in CSF were statistically different among the four groups. The Q_CXCL13_ in the neurosyphilis group were statistically higher than the syphilis/non-neurosyphilis (*P* = 0.001), and non-syphilis/non CNS infection group (*P* = 0.003)

The characteristics of the study participants were shown in Table 1. All of the enrolled subjects were negative for HIV. The concentrations of CXCL13 in the serum (χ^2^ = 72.115, *P* < 0.001) and CSF (χ^2^ = 22.653, *P* < 0.001) and Q_CXCL13_ (χ^2^ = 15.110, *P* = 0.001) were different among the neurosyphilis group, the syphilis/non-neurosyphilis group, the non-syphilis group and the healthy volunteers. The serum CXCL13 level was significantly higher in the neurosyphilis and syphilis/non-neurosyphilis groups than in the healthy volunteers (χ^2^ = 48.491, *P* < 0.001; χ^2^ = 17.170, *P* < 0.001). But there was no different in serum CXCL13 level either between neurosyphilis group and syphilis group, or neurosyphilis group and non-neurosyphilis group. Furthermore, we have divided non-syphilis/other central nervous system diseases into 11 patients with viral or cryptococcal infection diseases and 15 patients with noninflammatory neurological disorders. The serum CXCL13, CSF CXCL13 and Q_CXCL13_ were analyzed again. The results showed that serum CXCL13 concentrations had no value to differentiate between neurosyphilis and other neurologic viral and cryptococcal infection diseases or other noninflammatory neurological disorders (Fig. 1a). The CSF concentrations of CXCL13 were remarkably increased in patients with neurosyphilis compared to patients with syphilis/non-neurosyphilis (χ^2^ = 21.802, *P* < 0.001) and patients with non-syphilis/non central nervous system (CNS) infection group (χ^2^ = 9.340, *P* = 0.009, Fig. 1b). The Q_CXCL13_ was higher in the neurosyphilis group than the syphilis/non-neurosyphilis (χ^2^ = 12.352, *P* = 0.002, Fig. 1c) and non-syphilis groups (χ^2^ = 11.528, *P* = 0.003, Fig. 1c).

**Table 1:** Characteristic of the study population

Characteristic value	Neurosyphilis (n = 40)	Syphilis/non-neurosyphilis (n = 31)	Non-syphilis		Healthy volunteers (n = 49)	*P*
			Other CNS infection (n = 11)	Non CNS infection (n = 15)		
	n (%)	n (%)	n (%)	n (%)	n (%)	
Male sex	30 (75.0)	18 (58.1)	6 (54.5)	6 (40)	28 (57.1)	0.158
Serum RPR ≥ 1:32	20 (50.0)	4 (12.9)	0	0	0	
Reactive CSF RPR	23 (57.5)	0	0	0	0	
	P_50_ (P_25_–P_75_)	P_50_ (P_25_–P_75_)	P_50_ (P_25_–P_75_)	P_50_ (P_25_–P_75_)	P_50_ (P_25_–P_75_)	
Age^a^ (years)	54 (46–58)	49 (39–59)	41 (29–52)	50 (24–70)	34 (28–47)	<0.001 ^f^
CSF WBC^b^ (10 × 10^6^/L)	25 (8–126)	7 (3–14)	53 (7–82)	5 (2–13)	ND	0.001 ^f^
CSF Protein (500 mg/L)	461.3 (351.2–676.9)	386.6 (323.6–523.9)	853.3 (266.0–1499.1)	478.5 (344.2–587.2)	ND	0.141
albumin quotient	6.0 (4.2–8.9)	5.3 (3.4–7.2)	9.0 (5.2–17.8)	8.8 (3.9–18.1)	ND	0.058
Serum CXCL13^c^ (pg/mL)	140.3 (82.5–200.5)	82.5 (55.0–136.7)	170.2 (158.4–188.7)	118.7 (96.0–188.7)	23.4 (15.0–37.4)	<0.001 ^f^
CSF CXCL13^d^ (pg/mL)^f^	10.1 (5.2–20.8)	0.4 (0–3.9)	5.4 (1.4–14.8)	0.1 (0–8.8)	ND	<0.001 ^f^
Q_CXCL13_^e^	9.3 (4.6–32.0)	0.6 (0–13.0)	3.9 (0.5–8.7)	0.1 (0–4.8)	ND	<0.001 ^f^

Characteristics of the study participants were shown in Table 1. Concentration of CXCL13 in serum , concentration of CXCL13 in CSF  and Q_CXCL13_ were statistical significace and different among neurosyphilis group, syphilis/non-neurosyphilis group, non-syphilis group and healthy volunteers. The serum CXCL13 level was significantly higher in neurosyphilis and in syphilis/non-neurosyphilis as compared to healthy volunteers (x^2^ = 48.491, *P* < 0.001; x^2^ = 17.170, *P *< 0.001). The CSF concentration of CXCL13 was remarkably increased in patients with neurosyphilis as compared to the other two groups of syphilis/non-neurosyphilis (x^2^ = 21.802, *P* < 0.001) and non-syphilis (x^2^ = 7.677, *P* = 0.002). The Q_CXCL13_ was higher in neurosyphilis as compared to syphilis/non-neurosyphilis (x^2^ = 12.352, *P* = 0.002) and as compared to non-syphilis (x^2^ = 9.335, *P* = 0.009). *ND* not detected

^a^Neurosyphilis versus healthy volunteers, *P* < 0.001; syphilis/non-neurosyphilis versus healthy volunteers, *P* = 0.008

^b^Neurosyphilis versus syphilis/non-neurosyphilis, *P* = 0.018; neurosyphilis versus non-syphilis/non CNS infection, *P* = 0.024; non-syphilis/other CNS infection versus non-syphilis/non CNS infection, *P* = 0.002

^c^Neurosyphilis versus healthy volunteers, *P* < 0.001; syphilis/non-neurosyphilis versus non-syphilis/other CNS infection, *P* = 0.022; syphilis/non-neurosyphilis versus healthy volunteers, *P* < 0.001; non-syphilis/other CNS infection versus healthy volunteers, *P* < 0.001; non-syphilis/non CNS infection versus healthy volunteers, *P* < 0.001

^d^Neurosyphilis versus syphilis/non-neurosyphilis, *P* < 0.001; neurosyphilis versus non-syphilis/non CNS infection, *P* = 0.009

^e^Neurosyphilis versus syphilis/non-neurosyphilis, *P* = 0.001; neurosyphilis versus non-syphilis/non CNS infection, *P* = 0.003

^f^Data was compared to different groups, the difference was statistically significant

**Fig. 2:**
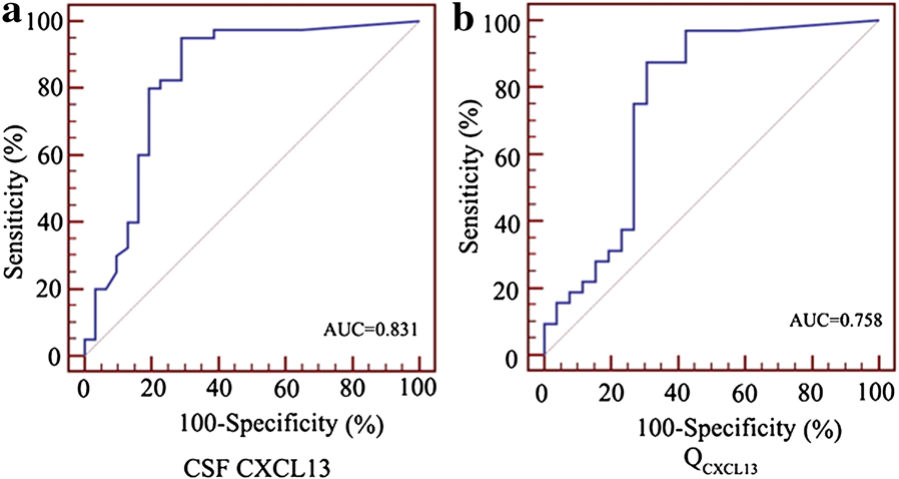
Receiver operating characteristics (ROC) curve analysis using CSF CXCL13 and Q_CXCL13_ for discriminating neurosyphilis from non-neurosyphilis/syphilis. **a** CSF CXCL13 yielded an AUC (the areas under the ROC curve) of 0.831 (95 % CI 0.724–0.938, *P* = 0.000) with 80.0 % sensitivity and 81.4 % specificity in discriminating neurosyphilis from non-neurosyphilis/syphilis. **b** QCXCL13 yielded AUC of 0.758 (95 % CI 0.620–0.895, *P* = 0.001) with 87.5 % sensitivity and 69.2 % specificity in discriminating neurosyphilis from non-neurosyphilis/syphilis

Furthermore, we explored the differences in the concentrations of CXCL13 in the serum and CSF and the Q_CXCL13_ among patients with different types of neurosyphilis. Based on its clinical manifestations, neurosyphilis can be divided into asymptomatic neurosyphilis, syphilitic meningitis, meningovascular neurosyphilis, general paresis, and tabes dorsalis. Of the 40 patients with neurosyphilis in the present study, 16 (40 %) had syphilitic meningitis, which accounted for the highest proportion of neurosyphilis, followed by general paresis (10, 25 %), meningo vascular neurosyphilis (6, 15 %), asymptomatic neurosyphilis (6, 15 %), and tabes dorsalis (2, 5 %). There was no significant difference in serum CXCL13 and CSF CXCL13 concentrations and the Q_CXCL13_ among patients with different types of neurosyphilis (Table 2).

**Table 2:** Serum and CSF CXCL13 concentrations in patients with different type of neurosyphilis

Type of NS	No %	Serum CXCL13 (pg/mL)	CSF CXCL13 (pg/mL)	CXCL13 index
Asymptomatic NS	15 % (6/40)	99.625 ± 51.757	10.458 ± 10.412	18.207 ± 14.652
Syphilitic meningitis	40 % (16/40)	166.034 ± 103.049	12.108 ± 8.129	71.767 ± 234.272
Meningovascular syphilis	15 % (6/40)	118.4201 ± 64.751	37.526 ± 70.852	62.473 ± 99.766
General paresis	25 % (10/40)	170.565 ± 93.286	33.405 ± 47.882	37.426 ± 55.628
Tabes dorsalis	5 % (2/40)	102.769 ± 81.190	17.804 ± 22.632	34.548 ± 42.221
χ^2^ value		5.226	0.334	3.190
*P* value		0.265	0.987	0.527

Kruskal–Wallis *H* test was used to determine differences among different types of neurosyphilis

### CXCL13 as a potential differentiation marker of neurosyphilis and non-neurosyphilis/syphilis

The diagnostic potential and discriminatory accuracy of CSF CXCL13 concentrations and the Q_CXCL13_ were evaluated by ROC curve analysis and the corresponding AUC values (Fig. 2). ROC analysis revealed that the CSF CXCL13 levels were robust in discriminating patients with neurosyphilis and non-neurosyphilis/syphilis, with an AUC value of 0.831 (95 % CI 0.724–0.938, *P* = 0.000). The highest accuracy was noted at a cut-off expression value of 4.871 pg/mL, where the sensitivity and specificity were 80.0 and 81.4 %, respectively. In addition, ROC curve analysis revealed that the Q_CXCL13_ can also serve as a valuable biomarker for differentiating neurosyphilis from non-neurosyphilis/syphilis, with an AUC of 0.758 (95 % CI 0.620–0.895, *P* = 0.001). At a cut-off value less than 2.408 for Q_CXCL13_, the sensitivity and specificity were 87.5 and 69.2 %, respectively.

### Correlations between CXCL13 and TPPA index/WBC in CSF

There was no correlation between TPPA index and CSF CXCL13 (r = 0.087, *P* = 0.593), and Q_CXCL13_ (r = 0.054, *P* = 0.752). It was same to CXCL13 and leucocyte count (r = 0.211, *P* = 0.191).

## Discussion

The diagnosis of neurosyphilis is still dependent on clinical and laboratory parameters that are not sufficiently sensitive. Scientists have made an effort to identify laboratory tests with high sensitivity and specificity for diagnosing neurosyphilis. In recent years, Marra et al. reported that CSF CXCL13 concentration may be particularly useful for diagnosing neurosyphilis in HIV-infected patients. Are these concentrations also useful in HIV-negative and with neurosyphilis patients? We retrospectively reviewed 97 inpatients from Zhongshan Hospital, Medical College of Xiamen University, China. Forty were HIV-negative and with neurosyphilis. In this study, the serum CXCL13 level was significantly higher in neurosyphilis and in syphilis/non-neurosyphilis patients than in healthy volunteers (χ^2^ = 48.491, *P* < 0.001; χ^2^ = 17.170, *P* < 0.001). However, there was no difference in serum CXCL13 levels among the neurosyphilis, syphilis/non-neurosyphilis, and non-syphilis patients. We have further divided non-syphilis/other central nervous system diseases into infectious group and without infectious group. The serum CXCL13, CSF CXCL13 and QCXCL13 were analyzed again. The results showed that serum CXCL-13 could not differentiate the neurosyphilis from other neurologic viral and cryptococcal infection diseases. Therefore, serum CXCL13 levels can’ t be used to support the diagnosis of neurosyphilis in HIV-negative patients.

However, we demonstrated significantly elevated levels of CSF CXCL13 in HIV-negative neurosyphilis patients compared to the other groups, which were similar to the observations conducted by Mothapo et al. (Mothapo et al. 2015). This article analyzed 103 syphilis patients with 47 non-HIV-infected patients. However, only six patients with neurosyphilis were also HIV-negative in their study, a number that was too small. In our study, we enlarged the study population. Was an excess of CXCL13 produced by monocytes in the CSF or the blood? Our study found that the mean albumin quotient was equal to or greater than 8.0 in neurosyphilis patients and non-syphilis neurological patients, which suggested that the blood brain barrier was damaged in these patients (Kowarik et al. 2012). Blood-CSF barrier dysfunction may lead *T. pallidum* invasion of the central nervous system, stimulating a local immune reaction and resulting in intrathecal synthesis of CXCL13 (Hytonen et al. 2014). Therefore, we investigated the Q_CXCL13_ to estimate the intrathecal synthesis of CXCL13, excluding the influence of blood CXCL13 contamination. The results showed that the Q_CXCL13_ in neurosyphilis patients was significantly higher than in syphilis/non-neurosyphilis patients, suggesting that intrathecal synthesis of CXCL13 did occur in HIV-negative neurosyphilis patients.

ROC analysis revealed that the CSF CXCL13 levels were robust in discriminating patients with neurosyphilis and non-neurosyphilis/syphilis, with an AUC of 0.831 (95 % CI 0.724–0.938, *P* = 0.000). The highest accuracy was at a cut-off value of 4.871 pg/mL, where the sensitivity and specificity were 80.0 and 81.4 %, respectively. The sensitivity and specificity in our study were higher than those in previous research (Marra et al. 2010). The proposed cut-off for abnormal CSF CXCL13 was low in comparison to similar studies regarding neuroborreliosis. Elevated CSF CXCL13 concentration has been shown to be a good marker for active Lyme neuroborreliosis (Hytonen et al. 2014; Bremell et al. 2013; Moniuszko et al. 2014; Wutte et al. 2014). The major findings of Rupprecht et al. study was that high levels of CXCL13 could be found in the CSF of patients with the two most common spirochetal CNS diseases, NB and NS, but the elevated CSF CXCL13 levels in patients with neurosyphilis were lower than in patients with neuroborreliosis (Rupprecht et al. 2007). There is substantial genotypic variation among Borrelia burgdorferi stains (Petzke and Schwartz 2015). The number of outer membrane proteins of Borrelia burgdorferi were more than *T. pallidum*. These may be the reason why the elevated CSF CXCL13 levels in neurosyphilis patients were lower than in neuroborreliosis patients. CXCL-13 positive results could be detected in patients with other neurological infectious diseases based on the cut-off for abnormal CXCL-13 in CSF (4.871 pg/ml). The neurological infectious diseases can be differentially diagnosed by microbiological and parasitological test, and brain imaging etc. However, *T. pallidum* causes syphilis and can only be cultured in vivo, and it is hard to detected in CNS directly. NS has various clinical manifestations, laboratory findings, magnetic resonance imaging and electroencephalogram findings, thus establishing the diagnosis is often difficult. There are few clinical criteria and no national guidelines available (gold standard) for NS diagnosis (Liu et al. 2013b). We found that CSF CXCL13 could also serve as a valuable supplementary biomarker for differentiating neurosyphilis from non-neurosyphilis/syphilis with HIV-negative patients. In Marra’s paper, she indicated that CSF CXCL13 ≥ 10 pg/mL could be used as a diagnostic indicators for symptomatic neurosyphilis characterized by high sensitivity (90 %) but low specificity (37 %) (Marra et al. 2010). The cut-off value for CSF CXCL13 in our study was lower than that in the study by Marra. This was mainly due to the different study population in the Marras’ article which comprised HIV-positive neurosyphilis patients. As we know, HIV can promote development of *T. pallidum* infection. Perhaps co-infection of *T. pallidum* and HIV promote the production of more CSF CXCL13 than *T. pallidum* infection alone. In Khutso’s study, authors used the positive CSF RPR as the gold standard for diagnosing neurosyphilis, on that criterion, only 6 out of 47 HIV-negative patients were neurosyphilis and included in HIV seronegative group. That is to say, the study subjects of HIV-negative neurosyphilis patients were too small to represent the CSF CXCL13 results of neurosyphilis patients (Mothapo et al. 2015). The elevated CSF CXCL13 levels were also indicated in Hu et al. report (2016) due to HIV and *T. pallidum* co-infection. In Hu et al.’s study, only 4 patients were diagnosed with neurosyphilis due to a positive CSF-VDRL (Hu et al. 2016). The number of neurosyphilis cases were also too small to reflect the real situation of CSF CXCL13 in the neurosyphilis. However, in our study, we mainly focused on HIV-negative patients, not only because the less population of co-infection of HIV and *T. pallidum* but also to rule out the impact of the HIV virus. The HIV-negative population in our research is the main difference from previous study. In addition, some scholars have indicated that the *T. pallidum* in different area was various in genotypes, so as to lead to differing in pathogenicity. For example, Wu et al. reported that subtype 14f/f (18 isolates) was the most common isolate in Taiwan, followed by 14 f/c (3), 14 b/c (3), and 14 k/f (Wu et al. 2012). Florindo et al. indicated that the predominant subtype in European countries, subtype was 14a, followed by 14d and 14f, which was different from the genotype in Taiwan (Florindo et al. 2008). This geographic distribution of different genotype of *T. pallidum* may also partly explain why our results are lower than those of other reports. Our further analyses revealed that the Q_CXCL13_ could also serve as a valuable biomarker for differentiating neurosyphilis from non-neurosyphilis/syphilis, with an AUC of 0.758 (95 % CI 0.620–0.895, *P* = 0.001). At the cut-off value less than 2.408 for the Q_CXCL13_, the sensitivity and the specificity were 87.5 and 69.2 %, respectively. The study results showed that the Q_CXCL13_ was more sensitive (87.5 vs. 80.0 %) than CSF CXCL13. Therefore, CSF CXCL13 and Q_CXCL13_ may be useful as markers for the diagnosis of neurosyphilis in HIV-negative patients.

In our study, the positive rate for the CSF RPR test was 55 %, similar to the literature reports of 50 % (Busl and Bleck 2013). According to the 2008 European Guidelines on the Management of Syphilis, a TPPA index >70 is most reliable in supporting the diagnosis of neurosyphilis, similar to the *T. pallidum* hemagglutination index (Mothapo et al. 2015). We had reported that the TPPA index significantly correlated with CSF WBCs. Thus, we investigated the correlation between CXCL13 levels and the TPPA index, as well as CXCL13 levels and CSF WBCs. The result showed that there was no correlation between CSF CXCL13 and CSF TPPA index/CSF WBCs. Therefore, our results suggest that intrathecal *T. pallidum* antibody production will not correlate with the induction of CXCL13. Because of the poor sensitivity of the CSF RPR and the low specificity of the CSF TPPA, CSF CXCL13 concentrations may serve as a supplementary biomarker of neurosyphilis. Does the CXCL13 concentration relate to the progression of neurosyphilis? To investigate whether there are particular types of neurosyphilis that are associated with CXCL13 production, we further analyzed the serum and CSF concentrations of CXCL13 in different types of neurosyphilis. There were no significant differences among them. This suggests that the central nervous system damage caused by various types of neurosyphilis has similar effects on the central nervous system.

To the best of our knowledge, this is the first article to analyze the relationship between HIV-negative neurosyphilis and the Q_CXCL13_, and is the first large sample analysis to describe the relationship between HIV-negative neurosyphilis and CXCL13 levels. Several limitations of our study should be acknowledged, such as the potential misclassification of different types of neurosyphilis patients with a prior history of syphilis due to inadequate medical records or hospital public health registries. Additionally, because healthy persons did not undergo an immediate lumbar puncture, we could not compare CSF results between the neurosyphilis group and the healthy controls.

In conclusion, when infection of *T. pallidum* occurs, CSF CXCL13 levels and Q_CXCL13_ may be potential markers for the diagnosis of neurosyphilis, as demonstrated by the elevated CSF CXCL13 levels in patients with suspected neurosyphilis. CSF CXCL13 levels and the Q_CXCL13_ are novel important promising biomarkers in differentiating neurosyphilis patients from non-neurosyphilis/syphilis patients without HIV infection.

## Abbreviations

CXCL13: chemokine ligand 13
ROC: receiver-operator characteristic
QCXCL13: CXCL13 quotient
CSF: cerebrospinal fluid
CSF-VDRL: cerebrospinal fluid-venereal disease research laboratory
RPR: rapid plasma reagin
TPPA: *T. pallidum* particle agglutination
WBC: white blood cell
ECLIA: electrochemiluminescence immunoassay
Q_alb_: albumin quotient
AUC: area under the curve
CNS: central nervous system
ND: not detected
